# A Small‐Scale Regional External Quality Assessment of Nucleic Acid Amplification Tests for *Bordetella pertussis* in China

**DOI:** 10.1002/jcla.70304

**Published:** 2026-07-28

**Authors:** Yuanyuan Guo, Junjiang Zhou, Zhijie Li, Jiangming Xiao, Tian Li, Changchun Niu, Yang Luo

**Affiliations:** ^1^ Department of Laboratory Medicine, Chongqing Center for Clinical Laboratory Chongqing Academy of Medical Sciences, Chongqing General Hospital, School of Medicine, Chongqing University Chongqing China

**Keywords:** *Bordetella pertussis* (BP), external quality assessment (EQA), nucleic acid amplification test (NAAT)

## Abstract

**Background:**

*Bordetella pertussis* (BP) infection remains a major public health concern because of its substantial clinical and epidemiological impact. Although nucleic acid amplification tests (NAATs) are widely used for pertussis diagnosis, their real‐world performance in routine clinical laboratories has not been systematically evaluated. This study implemented an external quality assessment (EQA) program to assess laboratory proficiency and identify potential sources of analytical variability.

**Methods:**

An EQA panel comprising five samples (four positive and one negative) was prepared using 10‐fold serial dilutions of BP (2.0 × 10^2^–2.0 × 10^5^ copies/mL). The panel was distributed to participating laboratories, and performance was evaluated based on qualitative agreement with the expected results.

**Results:**

A total of 69 laboratories participated, including 63 (91.30%) hospital‐based laboratories and 6 (8.70%) independent clinical laboratories, generating 345 test results. All laboratories achieved acceptable performance (scores ≥ 80). The overall concordance rate was 98.84% (341/345). Four false‐negative results were identified, all of which occurred in the lowest‐concentration sample (200 copies/mL).

**Conclusion:**

Pertussis NAATs demonstrated generally reliable diagnostic performance among participating laboratories in Chongqing, China. However, challenges remain in detecting low‐copy‐number specimens near the analytical sensitivity threshold. Continuous quality monitoring, standardized workflows, optimized nucleic acid extraction procedures, and regular performance verification are essential to ensure accurate pertussis diagnosis and support effective public health surveillance and outbreak response.

## Graphical abstract

An external quality assessment (EQA) was conducted to evaluate the performance of nucleic acid amplification tests (NAATs) for *Bordetella pertussis* across 69 laboratories in Chongqing, China. Among 345 total reports, 341 (98.8%) were concordant with expected results, while four false negatives occurred in low‐concentration samples near the analytical detection limit. These findings demonstrate overall reliable diagnostic capacity but highlight the need for continuous quality monitoring, optimized nucleic acid extraction, and standardized laboratory workflows to ensure accurate pertussis detection.

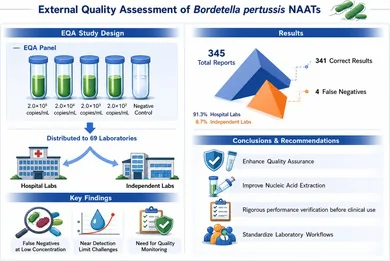

**Abbreviations**
BP:
*Bordetella pertussis*

Ct: cycle threshold
EQA: external quality assessment
NAAT: nucleic acid amplification test
NMPA: National Medical Products Administration
RT‐qPCR: real‐time quantitative polymerase chain reaction

## 1 Introduction

Pertussis, or whooping cough, is a highly contagious respiratory disease primarily caused by *Bordetella pertussis* , although related species such as *B. parapertussis* and *B. holmesii* may also contribute to infection [1, 2, 3]. Despite widespread vaccination, pertussis has re‐emerged globally in recent years and remains an important public health concern, particularly among infants and vulnerable populations [4, 5, 6]. Diagnostic delays, species misidentification, and limitations in current vaccination strategies may contribute to the continued circulation of pertussis pathogens [7, 8, 9]. Therefore, accurate and timely laboratory diagnosis is essential for disease control and outbreak management.

Current laboratory diagnostic methods for pertussis include bacterial culture, serological assays, antigen detection, and nucleic acid amplification tests (NAATs) [10, 11]. Among these approaches, NAATs are widely used because of their rapid turnaround time, high analytical sensitivity, and superior diagnostic performance compared with conventional culture methods. Real‐time quantitative polymerase chain reaction (RT‐qPCR) can detect low bacterial loads and differentiate *B. pertussis* from related Bordetella species using targets such as IS481 and ptxA‐pr [3, 12]. In China, several commercially available NAAT assays for the detection of *B. pertussis* have been approved for clinical use. However, the real‐world performance of these assays may be influenced by multiple factors, including specimen processing, nucleic acid extraction efficiency, amplification systems, and interpretation of results [13, 14, 15]. Ensuring the reliability and consistency of *B. pertussis* nucleic acid testing, therefore, remains critically important.

EQA is an important component of laboratory quality management and provides an objective approach for evaluating inter‐laboratory testing performance and identifying potential analytical variability [16, 17]. However, data regarding inter‐laboratory proficiency in *B. pertussis* nucleic acid testing remain limited in China. Therefore, we conducted a regional EQA program for *B. pertussis* nucleic acid amplification testing in Chongqing, China, to evaluate the diagnostic performance of participating laboratories and identify areas for quality improvement and standardization.

## 2 Materials and Methods

### 2.1 Sample Preparation

The EQA panel was prepared using materials provided by Shanghai ZJ Bio‐Tech Co. Ltd. (Shanghai, China). Inactivated clinical specimens were used as the source material, to which sample diluent, stabilizers, and preservatives were added to maintain homogeneity and stability during storage and distribution. All sample preparation procedures were performed under biosafety conditions to prevent contamination.

The concentrations of the target analytes were assigned using digital PCR (dPCR). A certified BP reference material (BNCC337541; 2.40 × 10^5^ copies/μL) was used for concentration calibration. Nucleic acid extraction was performed prior to dPCR analysis, and quantification was carried out using the QIAcuity Probe PCR Kit (QIAGEN; Cat. No. 250101, Lot No. 175025461) on the QIAcuity One 5plex digital PCR platform (QIAGEN; Serial No. 00738), according to the manufacturer's instructions. Each sample was analyzed in three independent replicates, and the target copy number was calculated by the QIAcuity software based on Poisson distribution statistics. The assigned reference concentration for each sample was defined as the mean of repeated measurements.

The final EQA panel consisted of five samples, including four positives and one negative control (Table 1). The positive samples were generated by serial 10‐fold dilutions of BP, with concentrations ranging from 2.0 × 10^2^ to 2.0 × 10^5^ copies/mL, thereby covering a broad dynamic range of pathogen loads for performance assessment. Each sample was aliquoted into 2 mL screw‐capped tubes and stored at −20°C until analysis.

**TABLE 1:** Panel composition of the EQA and the BP detection results.

Sample no.	Contents	Sample concentration (copies/mL)	Expected result	Specificity %	Sensitivity %
202521	*Bordetella pertussis*	2 × 10^2^	Positive	—	94.20 (65/69)
202522	*Bordetella pertussis*	2 × 10^4^	Positive	—	100 (69/69)
202523	Sterile saline	0	Negative	100 (69/69)	—
202524	*Bordetella pertussis*	2 × 10^5^	Positive	—	100 (69/69)
202525	*Bordetella pertussis*	2 × 10^3^	Positive	—	100 (69/69)

*Note:* Specificity %—No. of correct negative results/total no. of negative results. Sensitivity %—No. of correct positive results/total no. of positive results. The results for lot numbers 202524 and 202525 were derived from survey data.

### 2.2 Validation of the Panels

Before distribution to participating laboratories, the EQA samples were evaluated using six NMPA‐approved assays (Daan, XABT, Yilifang, Ustar, Biogerm, and Sansure) to confirm that the detection results were consistent with the expected values. In addition, the panel's homogeneity and stability were evaluated to ensure its reliability. These assessments were conducted at the Clinical Laboratory Center of Chongqing in accordance with the CNAS‐GL 003 guideline: Guidance on Evaluating the Homogeneity and Stability of Samples Used for Proficiency Testing [18]. Homogeneity was assessed by randomly selecting 10 samples from each batch, and stability was evaluated using three sets of 6 samples stored under different conditions (room temperature for 24 h, 2°C–8°C for 3 days, and −20°C for 1 month).

### 2.3 Organization and Performance of the EQA

The EQA panels were delivered to participating laboratories via cold‐chain courier to maintain sample integrity. For each EQA cycle, detailed instructions were provided regarding sample storage conditions, testing timelines, result reporting deadlines, assay requirements, and precautions for result submission. In addition, participating laboratories were requested to complete a questionnaire covering laboratory characteristics, nucleic acid extraction reagents and instruments, amplification platforms, extraction methods (manual or automated), claimed assay sensitivity, and other relevant laboratory practices.

Questionnaire data were collected and entered into Microsoft Excel for data cleaning and verification. Categorical variables were summarized as frequencies and percentages. The questionnaire results were mainly used for descriptive analysis of the testing methods and laboratory practices adopted by participating laboratories.

### 2.4 Results Evaluation

EQA results were evaluated based on qualitative concordance with the expected values. Test results that matched the expected outcomes were considered correct, whereas those that did not were considered incorrect. Each correct result was awarded 20 points, yielding a maximum possible score of 100 points per round (based on five samples). Performance was classified as “optimal” when all results were correct (100 points), “acceptable” when one result was incorrect (80 points), and “unacceptable” when more than one result was incorrect (score < 80 points).

### 2.5 Statistical Analysis

Statistical analyses were performed using Microsoft Excel (Microsoft Corp., Redmond, WA, USA) and SPSS version 19.0 (IBM Corp., Armonk, NY, USA). Data are presented as the mean ± standard deviation (SD). Comparisons between two groups were conducted using Student's *t*‐test, whereas comparisons among multiple groups were performed using one‐way analysis of variance (ANOVA). Homogeneity of variances was assessed using Levene's test. When equal variances were assumed, post hoc pairwise comparisons were conducted using the Bonferroni correction; otherwise, Welch's ANOVA was applied. A two‐sided *p‐value* < *0.05* was considered statistically significant. All figures were generated using GraphPad Prism version 8.0 (GraphPad Software, La Jolla, CA, USA).

## 3 Results

### 3.1 Validation of the EQA Panel

All EQA panel samples were evaluated for quality prior to distribution. Suitability testing of the five panel samples was conducted using multiple commercially available NAAT kits, including those from Daan, XABT, Yilifang, Ustar, Biogerm, and Sansure. These assays, which are primarily based on RT‐qPCR platforms, are routinely used in Chinese clinical laboratories. All samples containing BP were correctly detected as positive, whereas all samples containing sterile saline yielded negative results (Table 1).

In addition, duplicate testing of the homogeneity and stability samples was performed using the Biogerm assay. Homogeneity was evaluated by one‐way analysis of variance (ANOVA), and all calculated *F* values were below 3.02, indicating satisfactory sample homogeneity. Stability was assessed using the *t*‐test, and no statistically significant differences were observed among samples stored under different conditions (*p > 0.05*), confirming the stability of the panel samples (Tables S1–S4).

### 3.2 Participating Laboratories and Testing Methods

A total of 69 laboratories were enrolled in this EQA program. By the submission deadline, all 69 laboratories had returned results, totaling 345 reported results. Of these, 63 (91.30%) were hospital‐based clinical laboratories, while 6 (8.70%) were independent clinical laboratories.

Questionnaire data from all participating laboratories were successfully collected and analyzed. The survey showed that the majority of participating laboratories (62/69, 89.86%) employed automated magnetic bead–based nucleic acid extraction systems. In contrast, seven laboratories (10.14%) used manual lysis extraction reagents supplied with the PCR kits, according to the manufacturers' instructions. All laboratories utilized commercial BP PCR assays, including six assays approved by the National Medical Products Administration (NMPA). Among these, the Sansure assay was the most commonly used platform, followed by the Daan and Biogerm assays. The participating laboratories exhibited some variability in nucleic acid extraction methods and assay platforms, as summarized in Table 2.

**TABLE 2:** Overview of participating laboratories and assay configurations in the BP EQA program.

Laboratory types		Nucleic acid extraction methods		PCR assay platforms					
Hospitals	Companies	Automated magnetic bead	Manual lysis extraction	XABT	Sansure	Yilifang	Ustar	Daan	Biogerm
63 (91.3%)	6 (8.7%)	62 (89.86%)	7 (10.14%)	2 (2.9%)	44 (63.77%)	3 (4.35%)	1 (1.45%)	11 (15.94%)	8 (11.59%)

Abbreviations: Biogerm, Shanghai Bio‐Germ Medical Technology Co. Ltd. (Shanghai, China); Daan, Guangzhou Daan Gene Co. Ltd. (Guangzhou, China); Sansure, Sansure Biotech Co. Ltd. (Changsha, China); Ustar, Ustar Biotechnologies (Hangzhou) Co. Ltd. (Hangzhou, China); XABT, Beijing Applied Biological Technologies Co. Ltd. (Beijing, China); Yilifang, Shenzhen Yilifang Biotech Co. Ltd. (Shenzhen, China).

### 3.3 Performance of Participating Laboratories

Among the 345 reported results, 341 were fully consistent with the expected outcomes. According to the predefined scoring criteria, all participating laboratories achieved satisfactory performance, including 65 laboratories classified as “optimal” and 4 laboratories classified as “acceptable”. No laboratory received an unsatisfactory rating.

Only sample 202521 (200 copies/mL) showed a reduced detection rate, with a sensitivity of 94.20% (65/69) (Table 1). The four discordant results were all false negatives and occurred exclusively in this lowest‐concentration sample.

To further explore factors potentially associated with these false‐negative results, the characteristics of the assays used by the participating laboratories were examined. The key performance parameters of the six National Medical Products Administration (NMPA)‐approved assays and the assay platforms used by participating laboratories are summarized in Tables 3 and 4. Descriptive analysis indicated that the false‐negative results for sample 202521 were concentrated in two assay platforms. However, these observations should be interpreted cautiously because of the limited number of laboratories within the respective subgroups.

**TABLE 3:** Key performance parameters of the six NMPA‐approved assays.

Reagents	Methods	Target genes	Claimed LOD (copies/mL)	Accuracy (CV%)	Template input volume (uL)	Applicable instruments	Total PCR cycle no.	Positive result
A	RT‐qPCR	NA	10,000	—	4	ABI Prism 7500, Roche cobas z 480, Roche LightCycler480.	40	Ct ≤ 38
B	RT‐qPCR	NA	400	< 5%	10	ABI Prism 7500, Life Technologies QuantStudio 5, SLAN‐96P, MA‐6000.	45	Ct ≤ 40
C	RT‐qPCR	IS481 + IS1663	1000	≤ 5%	5	ABI Prism 7500, Roche cobas z480, SLAN‐96S.	40	Ct ≤ 38
D	RT‐qPCR	IS481	500	< 5%	10	ABI Prism 7500.	45	Ct ≤ 38
E	RT‐qPCR	BP485 + BP0283	200	< 5%	5	ABI Prism 7500, Roche LightCycler480II, DA7600.	40	Ct ≤ 38
F	Isothermal Amplification‐Real Time Fluorescence Assay	BP485	500	< 5%	NA	UC0102/UC0108.	NA	Tt ≤ 24

*Note:* A—Yilifang; B—Sansure; C—Biogerm; D—XABT; E—Daan; F—Ustar.

Abbreviations: NA, not available; RT‐qPCR, real‐time quantitative polymerase chain reaction.

**TABLE 4:** Performance of detection assays employed by participating laboratories.

	Number of participants	Agreement, % (No. of correct results/total no. of results)				
		202521	202522	202523	202524	202525
All assays	69	94.20 (65/69)	100 (69/69)	100 (69/69)	100 (69/69)	100 (69/69)
Yilifang	3	100 (3/3)	100 (3/3)	100 (3/3)	100 (3/3)	100 (3/3)
Sansure	44	95.45 (42/44)	100 (44/44)	100 (44/44)	100 (44/44)	100 (44/44)
Biogerm	8	100 (8/8)	100 (8/8)	100 (8/8)	100 (8/8)	100 (8/8)
XABT	2	100 (2/2)	100 (2/2)	100 (2/2)	100 (2/2)	100 (2/2)
Daan	11	81.82 (9/11)	100 (11/11)	100 (11/11)	100 (11/11)	100 (11/11)
Ustar	1	100 (1/1)	100 (1/1)	100 (1/1)	100 (1/1)	100 (1/1)

**FIGURE 1:**
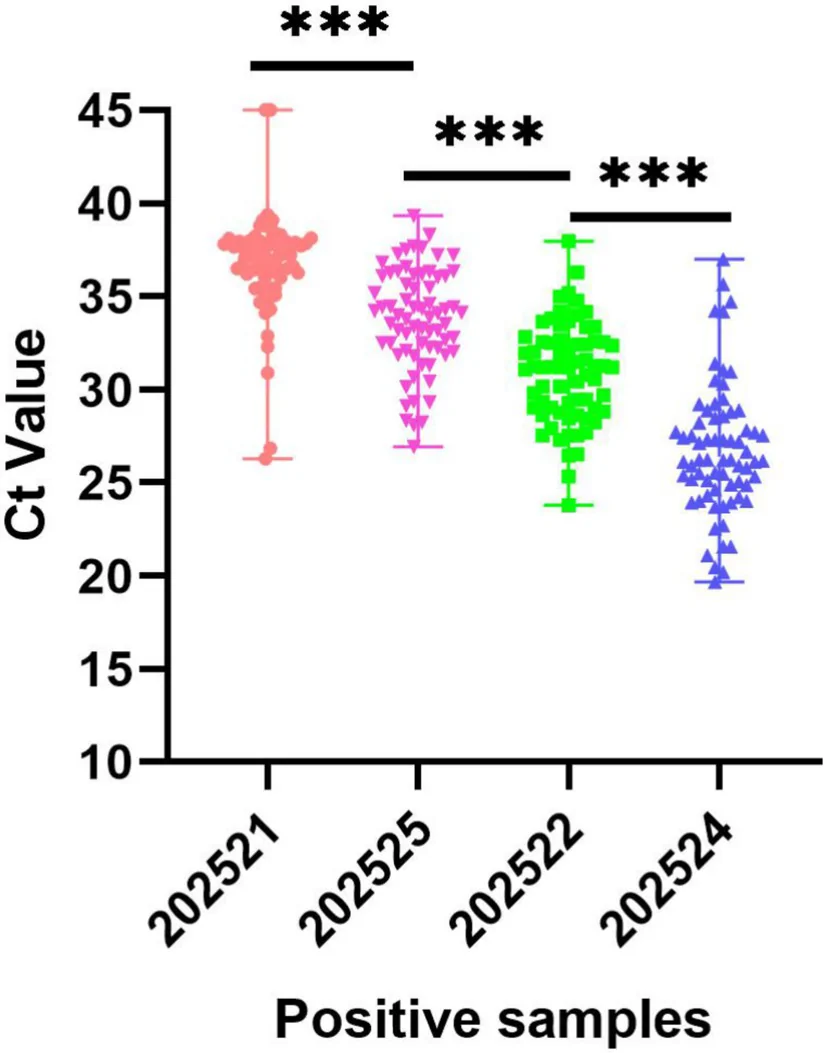
Distribution of Ct values for the four positive EQA samples (*n* = 69 laboratories per sample). Data are presented as mean ± SD. One‐way ANOVA demonstrated significant differences among the four groups (*p* < 0.001). Bonferroni‐adjusted post hoc comparisons showed statistically significant differences between all pairwise group comparisons ***p < 0.001.

In addition, analysis of the cycle threshold (Ct) values obtained from the four positive samples (Figure 1) showed a progressive decrease in Ct values with increasing target concentration, consistent with the expected concentration‐dependent amplification pattern. Significant differences were observed among the four sample groups by one‐way ANOVA (F [3, 272] = 147.166, *p < 0.001*), and Bonferroni‐adjusted post hoc analysis demonstrated significant differences between all pairwise comparisons (all *p < 0.001*). Similar results were obtained using Welch's ANOVA (F[3, 150.452] = 129.911, *p < 0.001*).

## 4 Discussion

The recent resurgence of pertussis in China has highlighted the importance of rapid and accurate laboratory diagnosis for both clinical management and public health control [19]. In response to increasing diagnostic demand, several commercial NAATs have been approved by the NMPA and are now widely used in clinical laboratories. However, variability introduced during specimen processing, nucleic acid extraction, amplification, and result interpretation may affect testing reliability [20]. Therefore, EQA programs are essential for assessing inter‐laboratory performance and supporting continuous quality improvement.

EQA panels are commonly prepared using a variety of well‐characterized materials, including inactivated or cultured clinical specimens, cell‐culture–derived reference strains, and commercial or lyophilized quality‐control samples [21, 22, 23]. In the present study, inactivated clinical specimens were selected as the primary source material and supplemented with appropriate diluents, stabilizers, and preservatives to better simulate real clinical matrices. This design enhanced the clinical relevance of the panel and allowed for a more realistic assessment of routine diagnostic performance. To ensure the accuracy of reference values, the concentrations of all samples were quantified using digital PCR. Notably, a low‐concentration sample (200 copies/mL) was deliberately included, as this level approaches the analytical sensitivity limits of many commercial NAAT assays. This strategy enabled us to specifically evaluate laboratory performance under near–limit‐of‐detection conditions, which represent a critical challenge in routine diagnostics. Moreover, the use of a 10‐fold serial dilution design generated predictable Ct intervals among positive samples, allowing not only assessment of qualitative detection ability but also evaluation of system linearity and Ct consistency across a wide dynamic range of pathogen loads. Collectively, this EQA design provides a practical framework for comprehensively assessing both sensitivity and analytical performance of pertussis NAATs in real‐world laboratory settings.

In this EQA activity, all participating laboratories achieved acceptable performance (scores ≥ 80), indicating that the overall diagnostic capability for *B. pertussis* nucleic acid testing is generally reliable across the surveyed institutions. Nevertheless, four false‐negative results were observed, all of which originated from the lowest‐concentration sample (200 copies/mL). Descriptive analysis further suggested that false‐negative results appeared to be more frequently observed in two assay platforms. Comparison of laboratory workflows revealed differences in the nucleic acid extraction methods used by these laboratories, and one of these false‐negative results was reported by a laboratory using a manual nucleic acid extraction method. However, because of the limited subgroup sizes, these findings should be interpreted cautiously.

This finding is not unexpected, as false‐negative results are more likely to occur when the pathogen load approaches the lower limit of detection (LOD) of the assay. First, the efficiency of nucleic acid extraction plays a critical role in diagnostic performance. Previous studies have demonstrated that extraction reagents based on manual lysis may contain detergents or organic solvents that interfere with downstream nucleic acid amplification. In contrast, automated extraction platforms can effectively avoid these issues, while also reducing operator‐dependent errors and minimizing the risk of cross‐contamination [24]. In addition, factors arising during pre‐analytical processing or analytical procedures—including sample degradation, improper specimen collection, and stochastic effects associated with very low copy numbers—may further contribute to detection failures [25, 26]. Previous molecular diagnostic evaluations and EQA studies have likewise shown that samples with low bacterial loads are more prone to reduced detection rates or false‐negative results [27, 28]. Collectively, these findings indicate that low‐copy‐number specimens represent a critical challenge in *B. pertussis* diagnostics and underscore the importance of continuous quality monitoring.

Another important consideration is the analytical sensitivity of the detection systems employed by the participating laboratories. As shown in Table 3, the six NMPA‐approved assays reported different claimed limits of detection, reflecting variability in analytical sensitivity across commercial platforms. Moreover, the analytical sensitivity achieved in routine clinical practice may not fully correspond to the manufacturers' claimed limits of detection owing to differences in assay design, nucleic acid extraction efficiency, amplification chemistry, and instrument performance across testing platforms. Such discrepancies could partly explain the false‐negative results observed for the lowest‐concentration sample in this EQA [29]. To ensure reliable diagnostic performance, laboratories are therefore strongly recommended to conduct thorough performance verification before implementing new assays, confirming that their analytical parameters meet the required clinical standards.

Furthermore, for specimens with low pathogen burden or rare targets, sensitivity may be improved through sample enrichment strategies prior to nucleic acid extraction [30, 31]. Approaches such as centrifugation to concentrate the cellular or bacterial fraction can increase the amount of target nucleic acid entering the extraction process, thereby enhancing the likelihood of detecting low‐copy *B. pertussis* . These measures may help mitigate the risk of false‐negative results in routine diagnostic practice, particularly when testing specimens near the assay's lower detection limit.

In our study, the consistent occurrence of false negatives at the lowest concentration suggests that while routine laboratory workflows are adequate for moderate‐to‐high pathogen loads, additional attention is required when processing specimens from early‐stage infections or partially treated patients, in whom bacterial burden is typically low. Strengthening quality control for nucleic acid extraction, improving operator training, and adopting higher‐sensitivity extraction or amplification platforms may help mitigate these risks.

Furthermore, the ability of laboratories to maintain relatively consistent Ct value intervals across the dilution series suggests that most detection systems exhibited acceptable amplification linearity within a certain dynamic range. However, it should be noted that Ct values are fundamentally intermediate parameters generated during the nucleic acid amplification process and represent method‐dependent indicators rather than directly standardized quantitative test results. Differences among testing platforms in target selection, amplification chemistry, nucleic acid extraction efficiency, instrument calibration, and threshold‐setting strategies may all contribute to variability in Ct values. Therefore, direct comparison of Ct values generated by different commercial detection systems may be misleading and should be interpreted with caution [32, 33]. This limitation should be considered when interpreting Ct‐based comparisons across laboratories or commercial platforms.

In contrast, expressing nucleic acid testing results using standardized quantitative units, such as copies/mL, may improve inter‐laboratory comparability and better align with the concentration characterization of EQA samples and the limits of detection specified by manufacturers. Such a standardization strategy may facilitate a more consistent assessment of the analytical sensitivity and overall performance of different testing platforms.

In conclusion, this small‐scale regional EQA demonstrated that most participating laboratories in Chongqing, China, achieved acceptable performance in *Bordetella pertussis* nucleic acid testing. The findings suggest that currently used commercial NAAT systems are generally capable of providing reliable diagnostic results under routine laboratory conditions, while also highlighting the persistent challenges associated with detecting low‐copy‐number samples near the analytical sensitivity threshold. However, this study was limited to a regional EQA program with a relatively small number of panel samples and participating laboratories. Therefore, larger‐scale, multicenter EQA studies are warranted to further evaluate the performance characteristics of different detection systems in real‐world clinical settings. Continuous quality monitoring, standardized workflows, the use of high‐efficiency nucleic acid extraction methods, regular participation in EQA programs, and performance verification of detection systems remain essential for ensuring the reliability of pertussis NAATs and supporting effective public health responses.

## Author Contributions

Yuanyuan Guo, Changchun Niu, and Yang Luo participated in the research design and helped draft the manuscript. Jiangming Xiao and Tian Li contributed to data acquisition. Junjiang Zhou and Zhijie Li performed the statistical analysis. Yuanyuan Guo and Zhijie Li prepared figures and tables. All authors have accepted responsibility for the entire content of this manuscript.

## Funding

This study was supported by Noncommunicable Chronic Diseases‐National Science and Technology Major Project (2025ZD0551300, 2025ZD0551301), the Scientific Research Project of the Chongqing Municipal Bureau for Disease Control and Prevention (No. 2026JKXM039), and the Science and Technology and Health Commission Program of Chongqing (No. 2020FYYX157).

## Ethics Statement

The authors have nothing to report.

## Conflicts of Interest

The authors declare no conflicts of interest.

## Supporting information


**Table S1:** Sample homogeneity calculation table for proficiency testing (e.g., 202522 sample).
**Table S2:** Sample stability calculation table for proficiency testing (e.g., 202522 sample).
**Table S3:** Sample homogeneity calculation table for proficiency testing (e.g., 202521 sample).
**Table S4:** Sample stability calculation table for proficiency testing (e.g., 202521 sample).

## Data Availability

The data that support the findings of this study are available from the corresponding author upon reasonable request.
