# Supplementary material for: A Small‐Scale Regional External Quality Assessment of Nucleic Acid Amplification Tests for Bordetella pertussis in China
Source: J Clin Lab Anal. 2026 Jul 28:e70304. Online ahead of print. doi: 10.1002/jcla.70304 (PMC13412113; doi:10.1002/jcla.70304)

Table S1. Sample homogeneity calculation table for proficiency testing（e.g., 202522 sample).


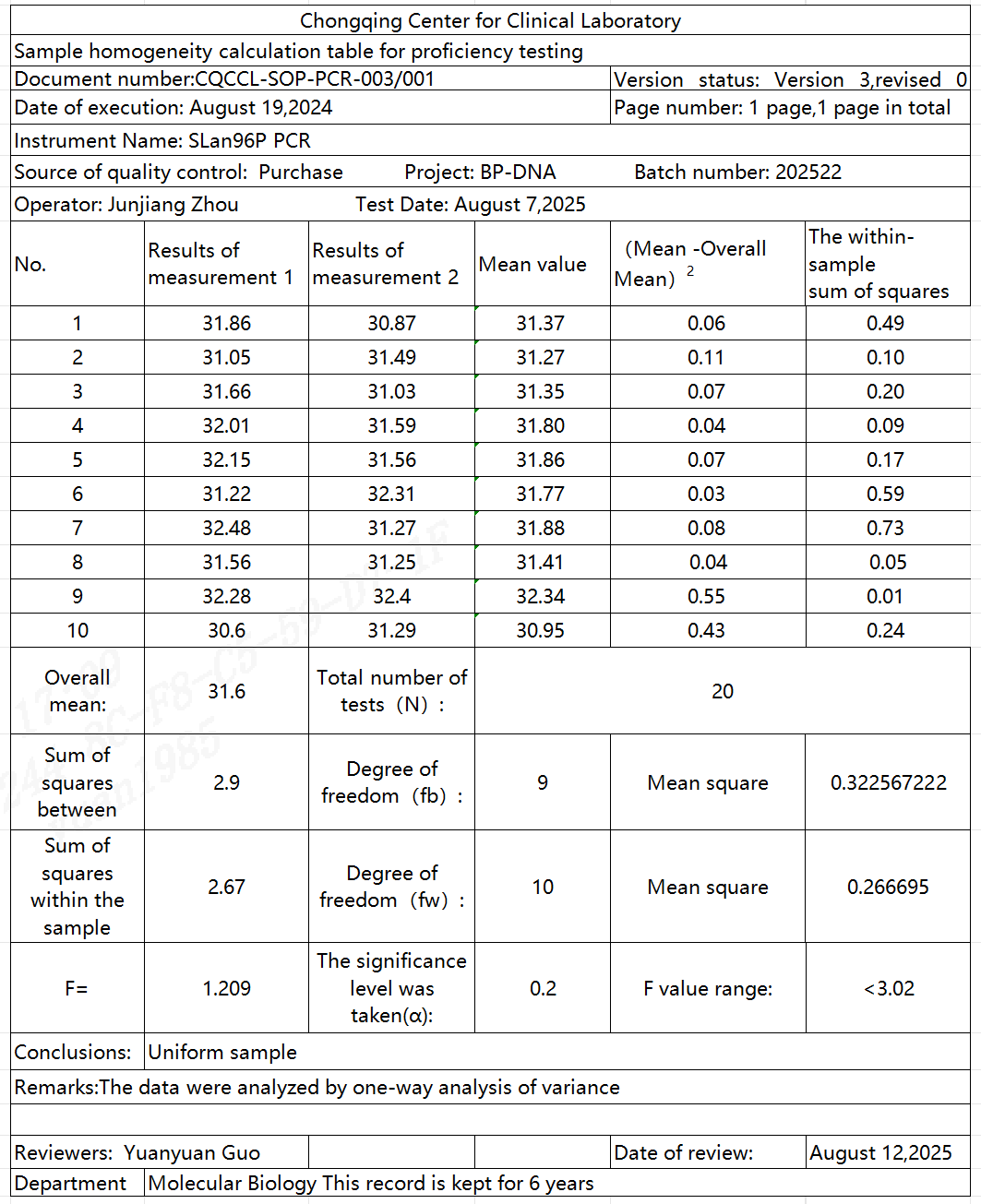


Table S2. Sample stability calculation table for proficiency testing（e.g., 202522 sample).


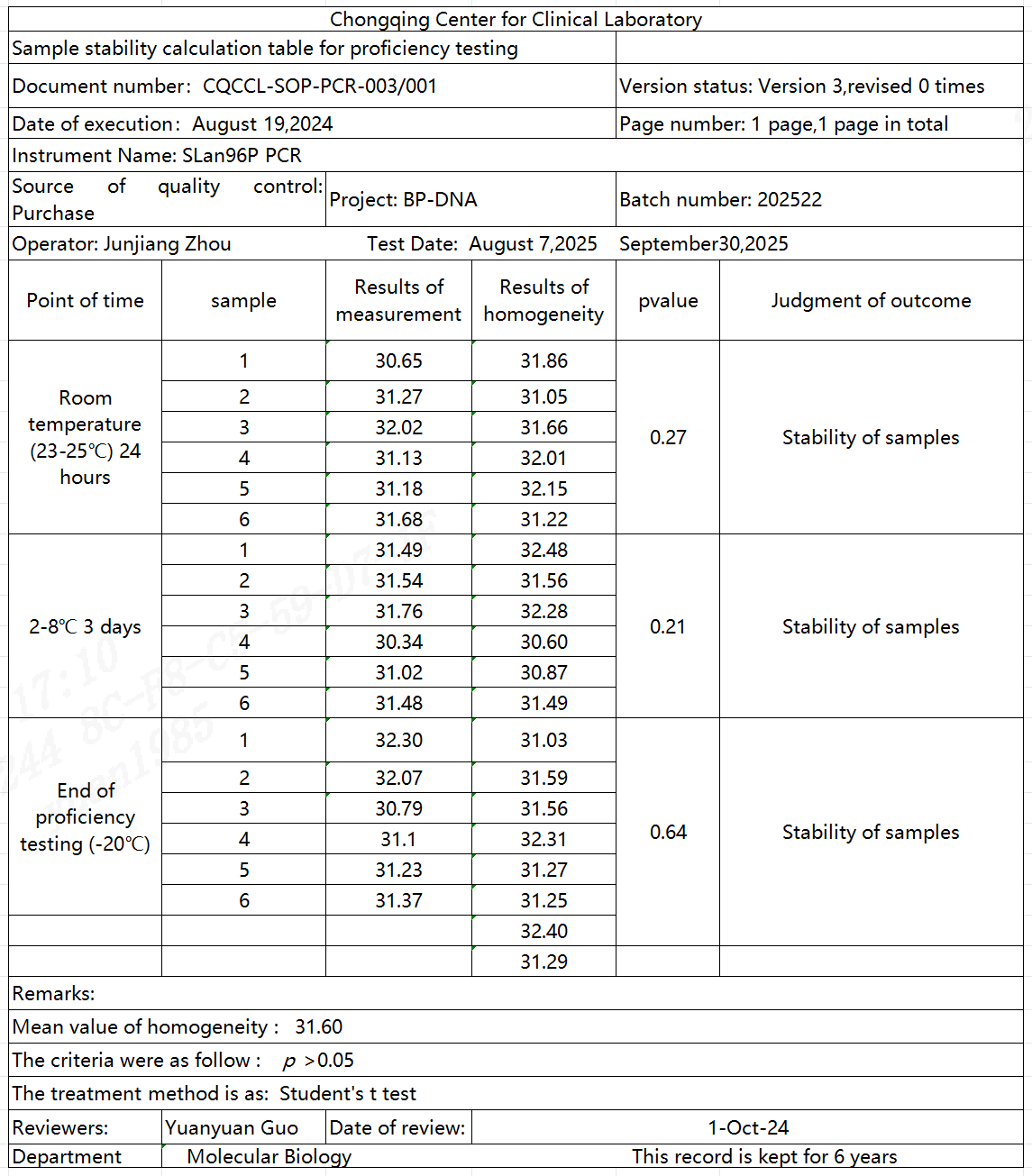


Table S3. Sample homogeneity calculation table for proficiency testing（e.g., 202521 sample).


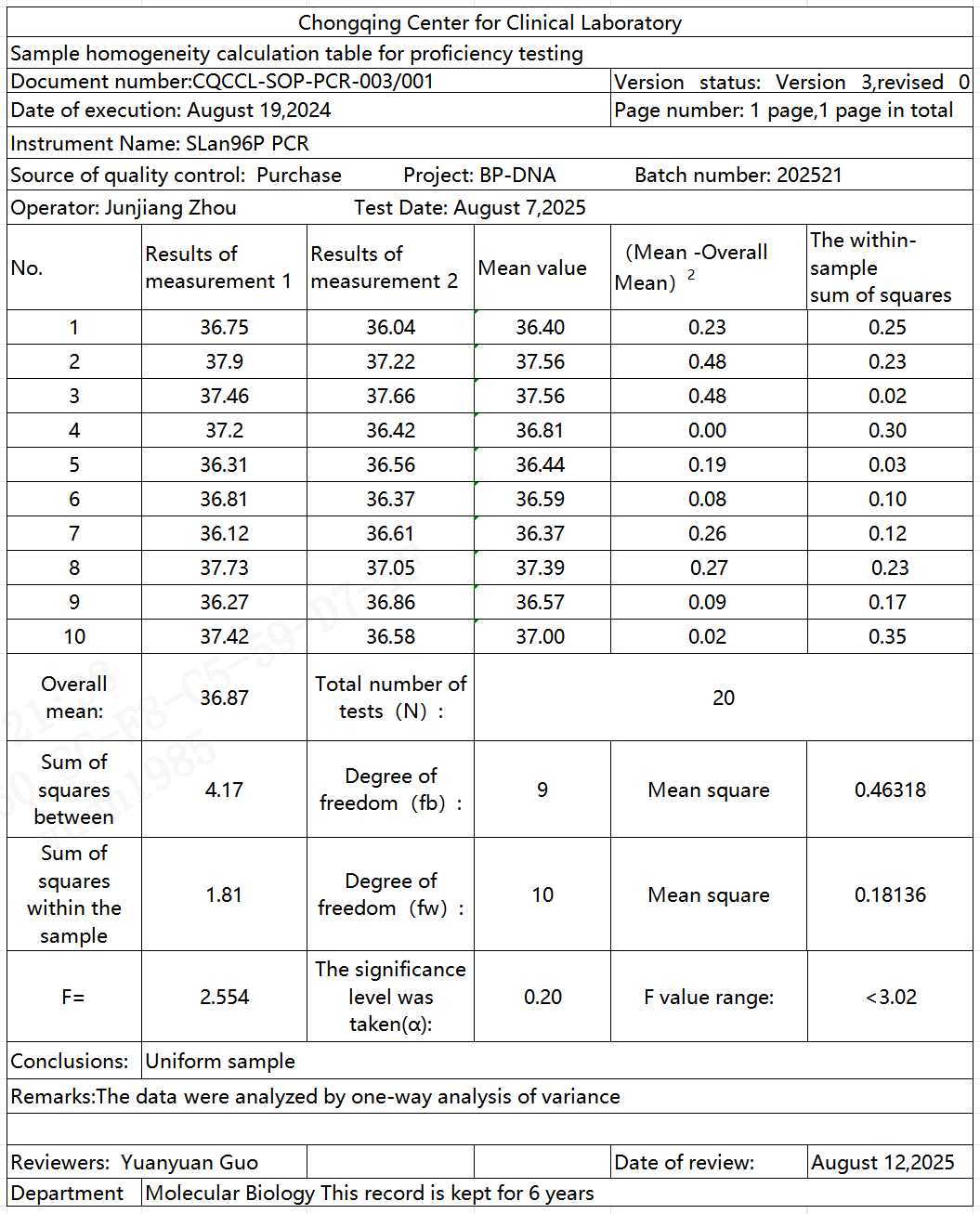


Table S4. Sample stability calculation table for proficiency testing（e.g., 202521 sample).


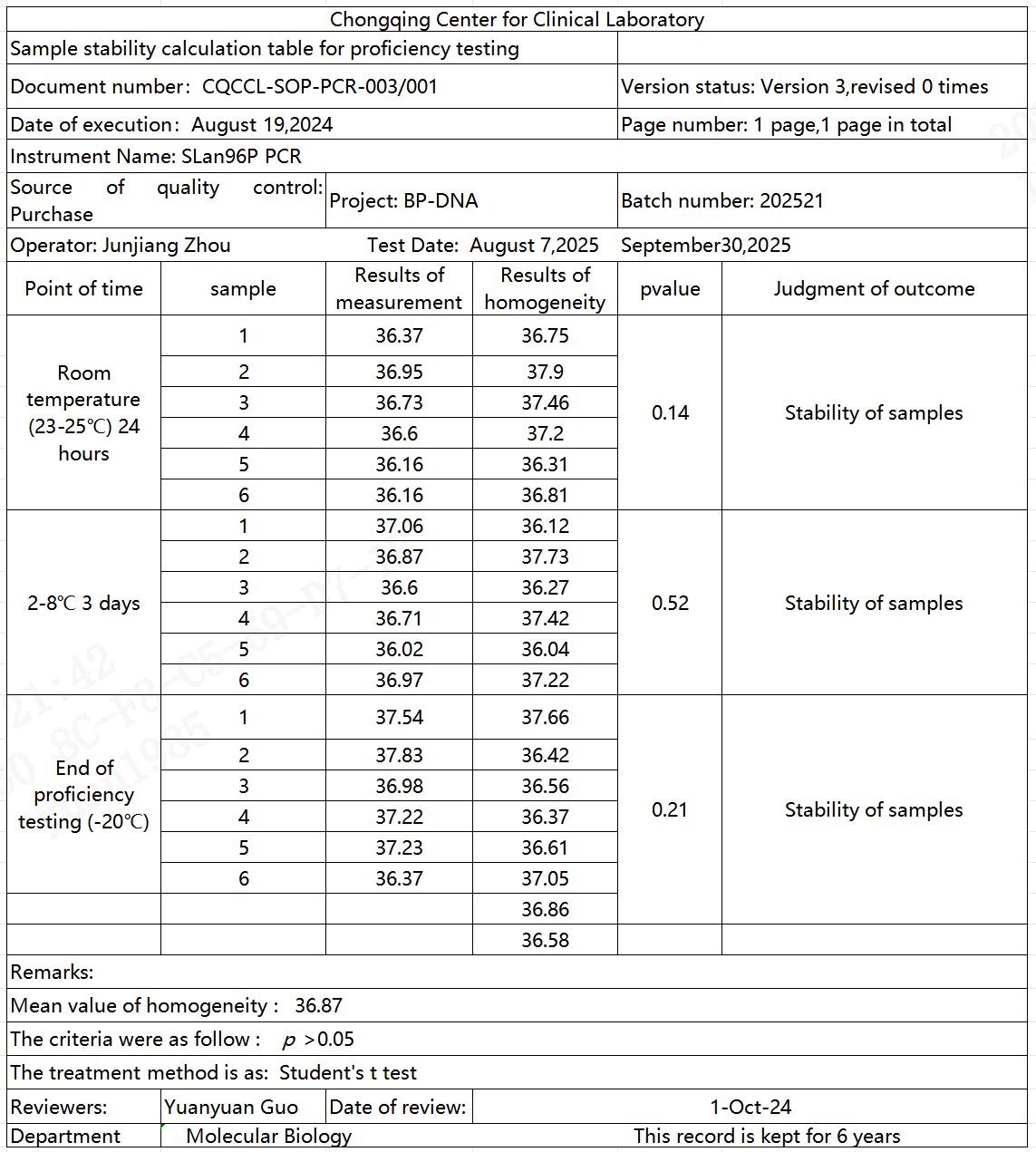

Supplement: Supplementary file 1 — Table S1: Sample homogeneity calculation table for proficiency testing (e.g., 202522 sample). Table S2: Sample stability calculation table for proficiency testing (e.g., 202522 sample). Table S3: Sample homogeneity calculation table for proficiency testing (e.g., 202521 sample). Table S4: Sample stability calculation table for proficiency testing (e.g., 202521 sample). [file JCLA-9999-e70304-s001.doc]
